# A Primary Care Emergency Service Reduction Did Not Increase Office-Hour Service Use: A Longitudinal Follow-up Study

**DOI:** 10.1177/2150132719865151

**Published:** 2019-07-29

**Authors:** Mika Lehto, Katri Mustonen, Jarmo Kantonen, Marko Raina, Anna-Maria K. Heikkinen, Timo Kauppila

**Affiliations:** 1 City of Vantaa, Vantaa, Finland; 2 Department of General Practice, University of Helsinki, Helsinki, Finland; 3 University of Tampere, Tampere, Finland

**Keywords:** community health centers, emergency department, primary care, practice management, mortality

## Abstract

This study, conducted in a Finnish city, examined whether decreasing emergency department (ED) services in an overcrowded primary care ED and corresponding direction to office-hour primary care would guide patients to office-hour visits to general practitioners (GP). This was an observational retrospective study based on a before-and-after design carried out by gradually decreasing ED services in primary care. The interventions were (*a*) application of ABCDE-triage combined with public guidance on the proper use of EDs, (*b*) cessation of a minor supplementary ED, and finally (*c*) application of “reverse triage” with enhanced direction of the public to office-hour services from the remaining ED. The numbers of visits to office-hour primary care GPs in a month were recorded before applying the interventions fully (preintervention period) and in the postintervention period. The putative effect of the interventions on the development rate of mortality in different age groups was also studied as a measure of safety. The total number of monthly visits to office-hour GPs decreased slowly over the whole study period without difference in this rate between pre- and postintervention periods. The numbers of office-hour GP visits per 1000 inhabitants decreased similarly. The rate of monthly visits to office-hour GP/per GP did not change in the preintervention period but decreased in the postintervention period. There was no increase in the mortality in any of the studied age groups (0-19, 20-64, 65+ years) after application of the ED interventions. There is no guarantee that decreasing activity in a primary care ED and consecutive enhanced redirecting of patients to the office-hour primary care systems would shift patients to office-hour GPs. On the other hand, this decrease in the ED activity does not seem to increase mortality either.

## Introduction

Emergency departments (EDs) often become overcrowded and this overcrowding may worsen their functions.^1-5^ At least partially, this overcrowding is caused by those patients who enter EDs without requiring emergency medical actions.^1-5^ Improved guiding and enhanced access to office-hour primary care services has been suggested as one solution to that problem.^6-9^

In the city of Vantaa, the health authorities took three different actions to guide nonurgent patients away from the local primary care ED during the years 2004-2008: application of ABCDE-triage combined with public guidance on the proper use of EDs,^10^ cessation of a minor supplementary ED,^11^ and, finally, application of “reverse triage” with enhanced guiding of the public to office-hour services from the remaining ED.^12^ The strategy was that those patients who did not require doctor services in EDs would be guided to office-hour GPs in the local primary care by the primary care system itself. We made a long-term study to examine whether this redirection to office-hour GPs really took place after the performed ED interventions and whether these interventions increased the workload of office-hour GPs. To study whether these interventions caused putative lethal side effects, we also studied mortality rates in different age-groups during the same follow-up period.

## Materials and Methods

### Study Design

The present work is a retrospective longitudinal quasi-experimental study with a before-and-after design in the primary care of the third largest city of Finland. This study was performed in Vantaa city, where in 2014 there were about 210 000 inhabitants. As everywhere in Finland, primary care is non-profit and municipalities, which fund this activity with taxes, maintain it. The ED system had 2 departments. The first evaluation was usually performed by the primary care ED system and if treatment in secondary care was necessary the patients were referred to the ED of the university clinic of Helsinki University (HUS) in the Peijas or Meilahti hospitals. Thus, the low acuity patients came first to the primary care ED system of the city of Vantaa.^10-12^

### ED Interventions

There were 3 different interventions performed in the primary care ED system of Vantaa city. Strategically, they were planned simultaneously but carried out gradually by the administration of the primary care of the city of Vantaa. At first, an ABCDE-triage system combined with public guidance on the proper use of EDs was applied in the main primary care ED of the city of Vantaa 1.1.2004.^10^ Briefly, this meant that a triage nurse evaluated all incoming patients and those patients who were evaluated not to have a need for emergency services (group E) were shown to a doctor after the more urgent patients (groups A, B, C, and D) were examined. This was combined with guidance about which problems were treated by emergency and which by office-hour GPs. Consequently, those patients who judged by themselves that their condition did not require emergency actions did not arrive at the ED when they realized that they would be forced to wait a long time to see a doctor. Second, a small suburban supplementary ED was closed in the western part of Vantaa city.^11^ Briefly, this meant that patients who originally sought help from a small nearby ED had to travel an average of 17 km more to reach the main ED compared with before the closure. Consequently, those patients who judged by themselves that it was not worth the extra burden of travelling to the remaining main ED to get their health problem treated immediately in emergency did not appear in the ED at all. Thirdly and finally, a tight “reverse triage,” based on ABCDE-categorization was applied in the remaining primary care ED.^12^ This meant that those patients who were triaged to group E did not meet a doctor in the ED. Instead they were given self-treatment advice or instructed in how to book a time with their own office-hour GP. Consequently, those patients who judged by themselves that their issue did not require emergency actions did not arrive at the ED when they realized that they would not see a doctor but would be sent away to office-hour services. In practice, these interventions led to a situation where the amount of doctor visits in the primary care ED system decreased by almost 50%.^10-12^

### Study Measures and Outcomes

The data were obtained from Graphic Finstar patient chart system (GFS, Logica LTD, Helsinki, Finland). The report generator of the GFS-system provided monthly figures for the total number of GP visits in Vantaa primary care. It was the main measure for analysis in the present study. Other measures were monthly deaths in different age groups (0-19, 20-64, and 65+ years), which data were provided by Statistics Finland. Data about size of population and number of GPs in Vantaa were provided by the statistics of Vantaa City. The preintervention period was from January 2002 to December 2007. The postintervention period started when the last ED intervention was implemented (January 2008) and it continued till the end of the follow-up (December 2014).

### Ethical Considerations

The register keepers (the health authorities of Vantaa) and the scientific ethical board of Vantaa City (TUTKE) granted permission (VD/8059/13.00.00/2016) to carry out the study. This study was made directly from the patient register without identifying the patients or doctors. According to the Finnish laws considering register studies (https://rekisteritutkimus.wordpress.com/luvat-ja-tietosuoja/) the study participants do not need to sign a Statement of Informed Consent because the study was retrospective, based on patient charts and the investigators did not contact the subjects.

### Statistical Analysis

To study when the ED interventions started to reduce the number of monthly GP visits nonparametric one-way repeated measurement analysis of variance (RM-ANOVA) followed by Dunn’s test^10-12^ was applied. *P* < .05 was considered to be a statistically significant difference. The rate of change in numbers of visits to monthly office-hour GPs and mortality in different age groups were analyzed using regression analysis followed by *t*-test (GLM procedure of SigmaPlot 10.0 Statistical Software, Systat Software Inc, Richmond, CA, USA).^13-15^ Thus, the GLM- model allowed us to count the mean slope (cofactor a) of the development of the amount of the GP visits (visits/month) and its standard error of the mean (SEM) before and after the interventions were performed. The comparisons with t-test were then performed between the slopes of these pre- and postintervention periods.

## Results

**Figure 1.**
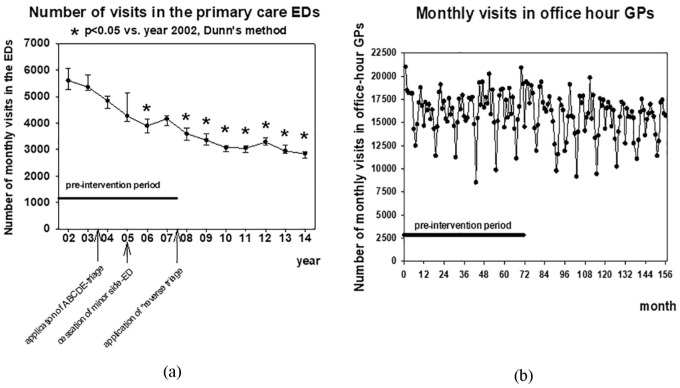
(a) Number of monthly visits to the doctors of the primary care emergency departments (EDs) in each year of the follow-up. Median (dot), 25% (lower bracket) and 75% (upper bracket) ranges are shown. (b) Number of monthly visits to the office-hour general practitioners (GPs).

When the whole follow-up period was taken into account in the analysis there was a constant and statistically significant decrease in the monthly number of visits to GPs in EDs (−18.830 ± 0.750 visits/month [mean ± SEM], *P* < .001, Figure 1a). The ED interventions started to induce a decrease in the number of monthly visits to EDs in 2006, after cessation of minor suburban supplementary ED. However, a constant decrease was obtained in 2008 and thereafter when the third and final intervention, for example, “reverse triage” was applied.

When the whole follow-up period was taken into account in the analysis, there was a constant and statistically significant slight decrease in the monthly number of office-hour visits to GPs (−11.085 ± 4.213 visits/month, *P* < .01, Figure 1b). However, when analyzed separately, there was no statistically significant change in the number of these visits in preintervention (−1.786 ± 13.544 visits/month) or postintervention (−17.260 ± 10.664) periods and the rates of change in these 2 periods did not differ from each other statistically significantly.

**Figure 2.**
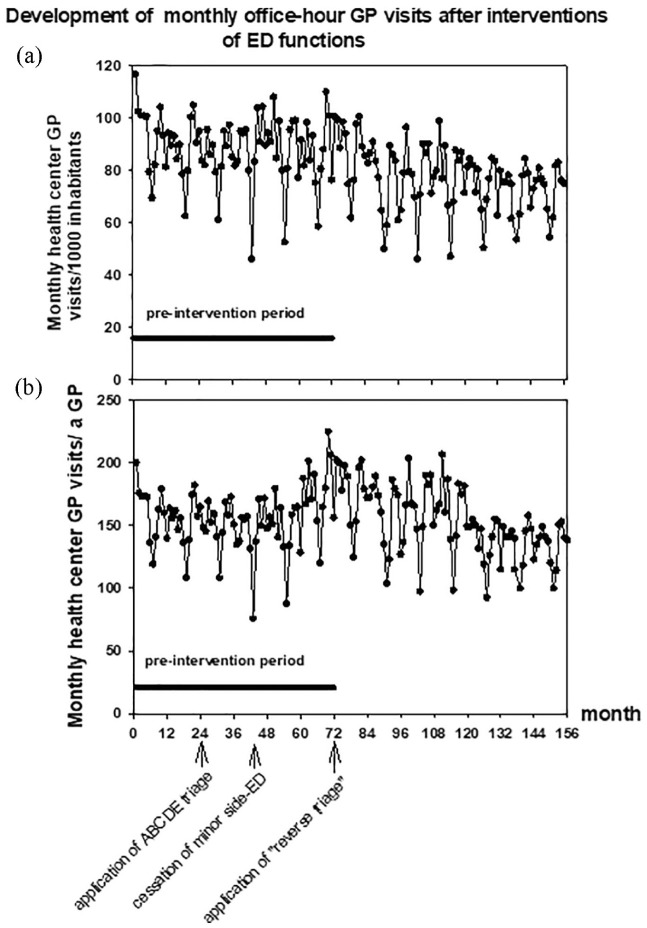
(a) Number of monthly visits to the office-hour general practitioners (GPs) adjusted to the population. (b) Number of monthly visits to the office-hour GPs adjusted to the number of GPs.

When the whole follow-up period was taken into account in the analysis, there was a constant and statistically significant slight decrease in the monthly number of office-hour visits to GPs/1000 inhabitants (−0.143 ± 0.0219 visits/1000 inhabitants in a month, *P* < .001, Figure 2a). There was no statistically significant decrease in the number of monthly visits to office-hour doctors per 1000 inhabitants during the preintervention period (−0.0850 ± 0.0732) but, as the number of inhabitants increased during the follow-up (Table 1), the number of monthly visits to office-hour doctors decreased statistically significantly in the post-intervention period (−0.176 ± 0.0535, *P* < .001, Figure 2a). However, the difference between the changes in the rate of decrease of these monthly visits before and after interventions was not statistically significant.

**Table 1.** Numbers of Inhabitants and General Practitioners (GPs) in the City of Vantaa.

Year	Number of Inhabitants	Number of GPs
2002	179 856	105
2003	181 890	105
2004	184 039	104
2005	185 429	113
2006	187 281	113
2007	189 711	93
2008	192 522	96
2009	195 397	94
2010	197 636	94
2011	200 055	96
2012	203 001	111
2013	206 705	111
2014	209 451	114

The number of office-hour GPs in the city of Vantaa remained roughly at the same level during the study period (Table 1). When the amount of office-hour visits was studied in relation to available GPs (monthly visits/GP) there was a decrease in the number of visits to office-hour GPs per GP (−0.114 ± 0.0466 visits/GP in a month, *P* < .05) during the whole follow-up period. There was no change in the preintervention period (0.111 ± 0.141) but in the postintervention period the number of these visits started to decrease (−0.614 ± 0.108, *P* < .001, Figure 2b). The difference between the changes in the numbers of these monthly visits per GP before and after interventions was statistically significant (*P* < .001).

**Figure 3.**
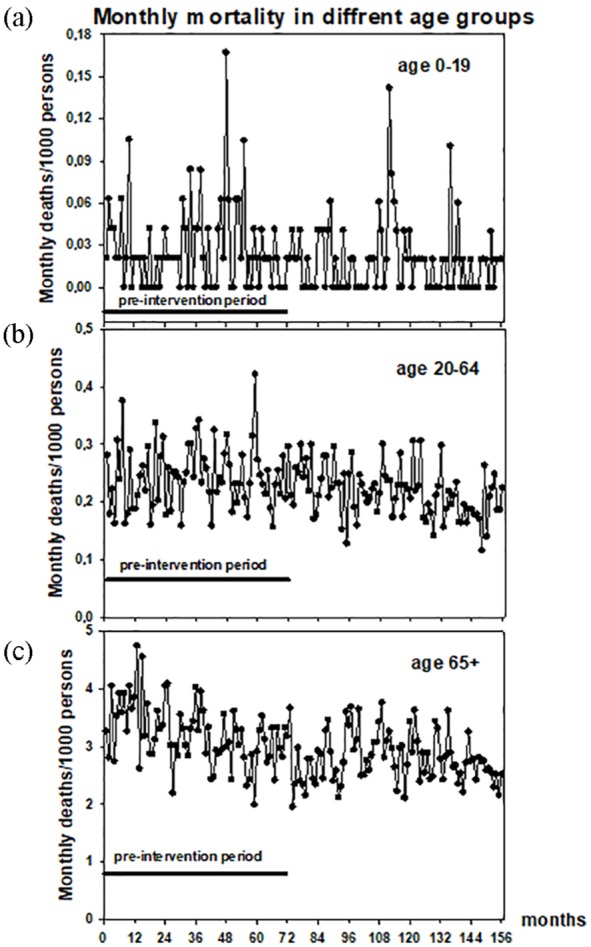
Numbers of deaths/1000 person in different age-groups: (a) 0-19 years, (b) 20-64 years, and (c) 65+ years.

There was no statistically significant change in total monthly mortality in preintervention (0.0000442 ± 0.000336 deaths/1000 in a month) or postintervention periods (−0.0000536 ± 0.000110). These rates of change did not differ statistically significantly from each other, either. This held true also with 0- to 19- and 20- to 64-years-olds (Figure 3a and b). The monthly number of deaths in age-group 65+ decreased slightly during the preintervention period (−0.0107 ± 0.00279, *P* < .001) but plateaued during the postintervention phase (−0.00179 ± 0.00190, Figure 3c). The difference between these rates was statistically significant (*P* < .01).

## Discussion

The number of visits to office-hour GPs decreased during the follow-up. This decrease continued after ED interventions. This was associated with decreased activity in GPs meeting patients after the ED interventions were performed. These changes did not, however, cause increased mortality.

In response to the suggestion that decreasing overcrowding would be beneficial,^6-9^ the administration of the primary care aimed to decrease the access of nonurgent patients to the ED system by making entrance to ED doctors more difficult and actively directing patients away from the EDs and to the office-hour GP services. The interventions used were able to reduce the visits to the primary care EDs by almost 50%.^10-12^ However, the present data suggests that redirecting the patients failed because the nonurgent patients did not visit office-hour GPs and, surprisingly, the rate of use of office-hour GPs decreased during the whole follow-up period. The reason for this decrease is unknown because the population of Vantaa city increased during the follow-up (Table 1) and, according to the official Finnish statistics, the population of Vantaa aged significantly during the follow-up period.^16^ A very small proportion of the patients turned to private sector GPs as the access to the EDs became difficult^11,12,17^ but despite this minor bypass flow there should not have been decreased demand for office-hour GP services in Vantaa. Nevertheless, the present interventions did not seem to result in excessive workload for the office-hour GPs. The number of patient visits per GP per se was not correlated with the number of GPs available. The number of GPs decreased in 2007 and the number of visits per GP started to decrease about a year after that. However, the number of visits did not return to the levels of 2002, although the number of GPs did (Table 1).

There were some indications that the number of nurse visits in daytime services increased during the follow-up, but this could not be verified because reliable data about the office-hour visits to the nurses were not available before 2009. We cannot exclude the possibility that the decrease in the number of visits to primary care GPs during office-hours was at least partially attributable to increased nurse activity because no additional office-hour doctor resources (Table 1) were allocated. In a former study from another city, the use of ABCDE-triage in an ED, for example, one of the interventions used in the present work, redirected patients from doctors to nurses.^17^ Furthermore, a recent qualitative study^18^ and a small scale quantitative study^19^ suggested that number of visits to office-hour primary care doctors are decreasing in Finland. The present quantitative data support these results. However, this observed decrease in visits to GPs does not seem to be a trend in primary care of other Nordic countries such as Denmark^20^ or in neighbor country Estonia.^18^

At least part of the patients who decided not to seek help from the primary care ED-system just stayed home. Thus, the present data also supports the suggestion that EDs may have “customers of their own” who are not likely to use ordinary daytime primary health care services.^5^ Some might have judged their clinical situation by themselves and concluded that the burden of visiting the EDs outweighed the benefits and that other primary care services were similarly not worth the bother. This may even be a desired state from the point of view of the ED system. In nonurgent health problems staying home instead of coming to the EDs may be understandable because patients who decide by themselves to leave the ED without being examined by a doctor have been reported as not suffering increased mortality or excessive adverse events in the short term.^21^

In all levels of health care and public health, mortality is a definitive measure of safety.^21,22^ The present ED interventions and the observed decrease in office-hour GP activities did not increase mortality in any of the studied age-groups. Therefore, their net effect seems to support the view that the present ED interventions caused no long-term lethal side effects. In the 65+ age-group the was a modest change in the rate of change in this parameter: a decrease in the mortality plateaued just after the last ED intervention was applied. However, without having the data about overall monthly mortality in Finland in this age-group as a comparison, we cannot conclude that the present interventions might have caused this observed plateauing in this parameter. Nevertheless, mortality is a crude parameter to study and it is not sensitive to primary care interventions.^22^

One strength of this study is that the present retrospective setting led to a situation where the study subjects did not know that they were being studied. There were no other major changes in the primary care which could have explained the observed changes at the time. Thus, the present result reflects real clinical activity. Furthermore, the ED model in Vantaa was well suited for studying how redirecting nonurgent patients to office-hour services succeeded. Vantaa used a combined ED model where primary care doctors first took care of every unscreened patient entering the ED and, if they were not able to help but considered the patient to require urgent treatment from the doctors of secondary care, they referred the patient to the secondary care in the same facility.^23^ An extra nurse was employed for the triage and all the patients coming to the EDs were triaged.^24^ About 10% of the patients coming to the ED were triaged to class A.^24^ Only triage class A patients and patients with referral from their own doctors were directly guided to the secondary care doctors who were not supposed to see nonscreened, and therefore nonurgent, patients at all.^23^ So, the putative effects of all the interventions in the present study were seen only in the primary care ED system and were thus focused on nonurgent patients entering the local ED. Furthermore, the ED- and office-hour services were under the same governance, health administration of the primary care of the city of Vantaa. Thus, the stakeholder was the same in the both ED and office-hour primary care systems and therefore there were no conflicts of interests when attempting to redirect the patients from EDs to office-hour services.

As a limitation, the present results can directly be applied only to primary health care. Furthermore, a control city with a similar ED-system, demography, and size but without simultaneous ED changes would have strengthened our conclusions. However, data from such a city were not available at that time in Finland. Data about possible changes in patient material or changes in ways to manage practices and diseases were not available. These factors have considerable effect on the development of numbers of visits to GPs. Data concerning these putative changes could have been obtained if we had access to patient chart information of individual patients, but we did not have that access. Who visits in office-hour primary care services and why, and what is done to him or her, will be an area of future studies.

## Conclusions

Decreasing activity in a primary care ED does not necessarily shift these patients to office-hour GPs. On the other hand, this decrease in the ED activity does not seem to increase mortality.
